# Phenotypic Prediction: Linking *in vitro* Virulence to the Genomics of 59 *Salmonella enterica* Strains

**DOI:** 10.3389/fmicb.2018.03182

**Published:** 2019-01-09

**Authors:** Angelina F. A. Kuijpers, Axel A. Bonacic Marinovic, Lucas M. Wijnands, Ellen H. M. Delfgou-van Asch, Angela H. A. M. van Hoek, Eelco Franz, Annemarie Pielaat

**Affiliations:** National Institute for Public Health and the Environment, Bilthoven, Netherlands

**Keywords:** Bayesian approach, gastro-intestinal tract, phenotypic virulence, quantitative risk assessment, virulence genes, WGS

## Abstract

The increased availability of whole-genome-sequencing techniques generates a wealth of DNA data on numerous organisms, including foodborne pathogens such as *Salmonella*. However, how these data can be used to improve microbial risk assessment and understanding of *Salmonella* epidemiology remains a challenge. The aim of this study was to assess variability in *in vitro* virulence and genetic characteristics between and within different serovars. The phenotypic behavior of 59 strains of 32 different *Salmonella enterica* serovars from animal, human and food origin was assessed in an *in vitro* gastro-intestinal tract (GIT) system and they were analyzed for the presence of 233 putative virulence genes as markers for phenotypic prediction. The probability of *in vitro* infection, P(inf), defined as the fraction of infectious cells passing from inoculation to host cell invasion at the last stage of the GIT system, was interpreted as the *in vitro* virulence. Results showed that the (average) P(inf) of *Salmonella* serovars ranged from 5.3E-05 (*S.* Kedougou) to 5.2E-01 (*S.* Typhimurium). In general, a higher P(inf) on serovar level corresponded to higher reported human incidence from epidemiological reporting data. Of the 233 virulence genes investigated, only 101 showed variability in presence/absence among the strains. *In vitro* P(inf) was found to be positively associated with the presence of specific plasmid related virulence genes (*mig*-5, *pef*, *rck*, and *spv*). However, not all serovars with a relatively high P(inf), > 1E-02, could be linked with these specific genes. Moreover, some outbreak related strains (*S.* Heidelberg and *S.* Thompson) did not reveal this association with P(inf). No clear association with *in vitro* virulence P(inf) was identified when grouping serovars with the same virulence gene profile (virulence plasmid, Typhoid toxin, *peg* operon and *stk* operon). This study shows that the *in vitro* P(inf) variation among individual strains from the same serovar is larger than that found between serovars. Therefore, ranking P(inf) of *S. enterica* on serovar level alone, or in combination with a serovar specific virulence gene profile, cannot be recommended. The attribution of single biological phenomena to individual strains or serovars is not sufficient to improve the hazard characterization for *S. enterica.* Future microbial risk assessments, including virulence gene profiles, require a systematic approach linked to epidemiological studies rather than revealing differences in characteristics on serovar level alone.

## Introduction

Recent years have seen a concerted effort in the scientific community and the food industry to analyze molecular data related to foodborne pathogens and understand their relevance for public health. The development in Next Generation Sequencing (NGS) techniques (e.g., Taboada et al., 2017) and algorithms (pipelines) for data analysis (e.g., Davis et al., 2015; Katz et al., 2017) has resulted in more precise and also quicker outbreak investigations (Gilchrist et al., 2015; Jackson et al., 2016), source attribution and genomic epidemiology (Franz et al., 2014, 2016). In addition, the International Life Science Institute (ILSI) has started several initiatives to share the views of different stakeholders in the application of NGS data in food safety management. For example, the “Microbiological Food Safety Task Force Expert Group: the use of NGS-translation into practice”^1^ and ILSI were engaged in a “Workshop on NG MRA (Microbiological Risk Assessment), integration of omics data into risk assessment.” This resulted in four position papers that are published (*e.g.*, den Besten et al., 2017; Haddad et al., 2018). Most of these studies focus on surveillance and outbreak investigations. Research hardly addresses the application of omics data in microbial risk assessment (MRA). Some studies exist in which genomic analysis has led to novel insights in the ‘hazard identification’ step of the MRA framework. For example, Maury et al. (2016) identified novel virulence factors in *Listeria monocytogenes* by studying the genomes of several clinical strains. Pielaat et al. (2015) demonstrated the non-trivial computational complications when molecular data are used for MRA. Practical implications were illustrated in that paper with a case study: linking single nucleotide polymorphisms (SNP) data of *Escherichia coli* O157:H7 to phenotypic behavior.

*Salmonella enterica* serovars are among the major foodborne diarrhoeal disease agents worldwide (Havelaar et al., 2015). While some serovars (among more than 2500 in total) dominate the human disease incidence (i.e., Enteritidis and Typhimurium) others rarely cause disease but can be found in animals, food and/or the environment (European Food Safety Authority [EFSA] and European Centre for Disease Prevention and Control [ECDC], 2017). Although outbreaks associated with relatively rare serovars do occur (Brandwagt et al., 2018). Such an epidemiological situation of *Salmonella* serovars could be the reflection of differences in virulence, in exposure, or both. In this study the *in vitro* virulence as well as the genetic virulence profile of various *Salmonella* serovars were investigated. A total of 59 *S. enterica* strains (32 different serovars) were screened for the presence of 233 virulence genes of *Salmonella*. In addition, all strains were tested in an *in vitro* Gastro Intestinal Tract (GIT) model system to quantify infectivity by estimating a probability of infection (P(inf)) (Wijnands et al., 2017). Data analysis aimed to identify whether different *Salmonella* serovars have different phenotypic and/or genotypic characteristics; if different *Salmonella* strains of the same serovar share the same phenotypic and genotypic characteristics; whether the phenotypic result [P(inf)] for the different *Salmonella* strains can be explained by genotypic properties (presence/absence of genes); and to what extent the outcomes of these investigations can be related to surveillance data on human incidence.

## Materials and Methods

A short description of the experimental set-up and data analysis are given in section “Gastrointestinal Passage” and “GIT System Data Analysis” below. Earlier, preliminary analysis were presented at the 9th International Conference on Predictive Modelling in Food (Pielaat et al., 2016). In that study, bacterial concentrations (counts per ml) were used for further calculations without the conversion of the dilutions in the sequential steps of the GIT system. In the present study, dilutions have been accounted for and a thorough procedure was used for quantifying the gastrointestinal passage system, including a Bayesian framework for statistical analysis is described in Wijnands et al. (2017). Additional aspects specific to the experimental set-up and data analysis of the *Salmonella* strains in the present study are given below.

### Bacterial Strains

In total, 59 strains belonging to 32 different *Salmonella* serovars were used for the *in vitro* GIT experiments and NGS. Two additional strains of S. Enteritidis (broiler) were used for the NGS experiments (see Supplementary Appendix A). The strains were obtained from the National Institute for Public Health and the Environment (RIVM) in the Netherlands. The center for zoonosis and environmental microbiology (cZ&O) of the RIVM has build this *Salmonella* strain collection over the years from different research projects. The collection was now used to quantify variability among the strains in *in vitro* gastro intestinal tract characteristics in relation to genetic properties. Table 1 includes an overview of the various strain sources associated for the different serovars.

**Table 1:** Summary of results from the GIT system assay of the 32 *Salmonella* serovars.

ID	Serovar	Source	Mean	SD	2.5%	97.5%	ID	Serovar	Source	Mean	SD	2.5%	97.5%
1	Agona	Chicken product	1.44E-03	1.97E-03	1.52E-04	5.61E-03	17	Livingstone	Chicken product	2.02E-03	2.51E-03	2.28E-04	8.20E-03
2	Anatum	Chicken product	2.41E-02	3.03E-02	2.44E-03	9.95E-02	18	Minnesota	Chicken product	6.28E-04	7.57E-04	6.34E-05	2.55E-03
3	Banana	Broiler	7.99E-04	1.54E-03	7.21E-05	3.24E-03	**19**	Montevideo	Broiler, chicken product	1.40E-03	3.12E-03	6.65E-06	8.24E-03
**4**	Braenderup	2 Chicken product	2.36E-02	5.08E-02	2.74E-04	1.35E-01	**20**	Paratyphi B v Java	Chicken product, human	4.91E-02	9.29E-02	8.76E-04	2.76E-01
**5**	Brandenburg	Human, chicken product, pork (^∗^2)	6.58E-02	1.29E-01	4.76E-04	4.07E-01	**21**	Rissen	3 Pork (^∗^2)	3.81E-02	5.13E-02	5.70E-04	1.64E-01
6	Bredeney	Chicken product	1.04E-02	1.32E-02	1.16E-03	4.12E-02	22	Saintpaul	Broiler	2.56E-02	2.99E-02	2.80E-03	1.01E-01
7	Cerro	Broiler	6.25E-02	7.52E-02	6.54E-03	2.55E-01	23	Schwarzengrund	Chicken product	3.39E-04	4.68E-04	3.18E-05	1.44E-03
**8**	Enteritidis (SE)	Broiler, 2 chicken product, Human	2.77E-02	4.40E-02	4.08E-04	1.28E-01	24	Senftenberg	Broiler	2.68E-03	3.23E-03	2.92E-04	1.08E-02
9	Gold Coast	Chicken product	3.69E-02	4.21E-02	4.10E-03	1.51E-01	25	Tennessee	Chicken product	1.13E-03	1.48E-03	1.09E-04	4.80E-03
10	Hadar	Chicken product	2.78E-03	3.34E-03	3.12E-04	1.11E-02	**26**	Thompson	Food, chicken product, human	2.47E-02	3.52E-02	1.36E-03	1.10E-01
**11**	Heidelberg	Food (^∗^2), chicken product (^∗^3), human (^∗^3)	3.50E-03	3.09E-03	8.00E-04	1.11E-02	**27**	Typhimurium (STM)	2 Pork (^∗^2), pork, 3 human (^∗^2)	1.67E-01	3.98E-01	7.72E-05	1.10E+00
12	Indiana	Broiler	1.38E-02	1.59E-02	1.61E-03	5.42E-02	**28**	Monophasic STM (S1 1,4,[5],12:i:-)	2 Pork (^∗^2), pork, 2 human (^∗^2), human	6.17E-02	6.97E-02	6.53E-03	2.32E-01
**13**	Infantis	Broiler, chicken product	1.58E-03	2.30E-03	1.32E-04	6.93E-03	**29**	Virchow	Broiler. Human	1.25E-03	1.77E-03	8.99E-05	5.51E-03
14	Jerusalem	Broiler	1.94E-03	2.75E-03	2.05E-04	8.05E-03	30	Weltevreden	Broiler	3.76E-02	4.88E-02	3.93E-03	1.52E-01
15	Kedougou	Chicken product	5.32E-05	8.75E-05	3.14E-06	2.53E-04	31	Worthington	Broiler	2.16E-03	2.87E-03	2.39E-04	8.72E-03
**16**	Kottbus	Broiler, human	4,15E-02	8,09E-02	3.06E-04	2.38E-01	32	Yoruba	Broiler	1.10E-03	1.42E-03	1.15E-04	4.55E-03

*Each serovar includes one or more strains from various sources, which add up to the total of 59 different strains included in the present study. The mean, standard deviation (SD) and 95% C.I. (2.5% lower bound and 97.5% upper bound) for P(inf) are given at serovar level. The ID numbers in boldface refer to serovars for which more than one strain was tested in the GIT system. Posterior distributions of P(inf) of the individual strains were used to come to average statistics on serovar level. Statistics for serovars having a single test strain are based on the final 3000 MCMC samples for the individual strain (see also section “GIT System Data Analysis”). ^∗^Indicates the number of replicates in the GIT system with the strain from that specific source.*

### Gastrointestinal Passage (GIT)

**FIGURE 1:**
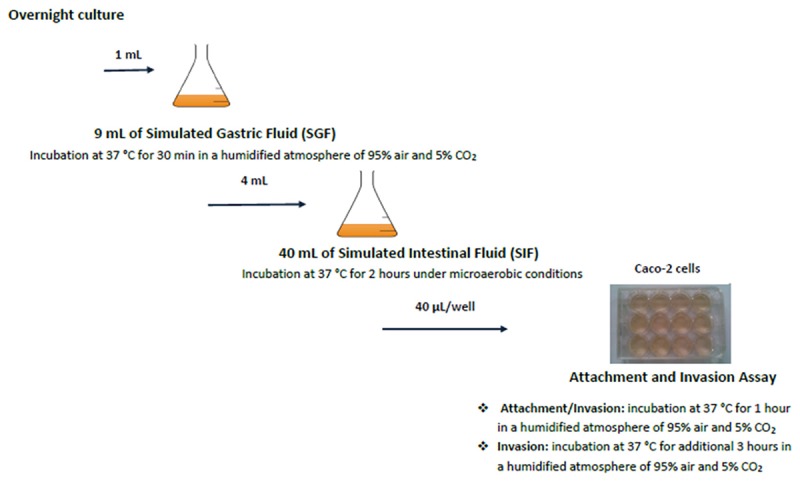
Schematic set-up of the simulated GIT-passage system. Replicate counts occur at all stages.

The different stages of the *in vitro* GIT-model simulating the human gastrointestinal tract are shown in Figure 1 (Wijnands et al., 2017). The GIT system is sequential, meaning that the *Salmonella* culture is transferred from one stage (SGF) to the next (SIF, ATT, and INV) without intermediate culturing.

For the GIT system, 1 ml overnight culture of *Salmonella* (ON) was mixed with 9 ml simulated gastric fluid (SGF). Samples (SGF-strain mixture) were incubated during 30 min at 37°C, after which 1 ml SGF-strain mixture was taken for microbiological analysis. From the remaining SGF-strain sample, 4 ml was mixed with 40 ml simulated intestinal fluid (SIF) and incubated during 2 h at 37°C. *Salmonella cells* surviving these SGF and SIF stages were incubated with fully differentiated Caco-2 cells to investigate their attachment (ATT) and invasion (INV) capability. This latter assay mimics the interaction with the small intestinal epithelium (Pinto et al., 1983; Hendricks et al., 1996). Caco-2 cells were obtained from the American Type Culture Collection (ATCC, HTB-37, United States). A 12-well plate (Corning, United States) was inoculated with 40 μl SIF-SGF-strain mixture per well, and incubated at 37°C during 1 h for the attachment assay (ATT). After aspiration of the medium, the cells were either lysed used for determining the number of attached and invaded *Salmonella*, or treated with gentamycin for the invasion assay(INV). For this invasion assay, the plates were incubated during 3 h at 37°C. The lysate was used for enumeration of the invaded *Salmonella.*

Surviving *Salmonella* cells were enumerated after every stage in the GIT model: ON, SGF, SIF, ATT, and INV (data not shown).

#### GIT System Data Analysis

A hierarchical Bayesian framework was used to analyze bacterial count data obtained from the gastro intestinal tract experiments. The methodology for this analysis has been described in Wijnands et al. (2017). In short, bacterial counts at any stage in the GIT system (ON, SGF, SIF, ATT, and INV) were assumed Poisson distributed. Concentrations were assumed lognormally distributed, and the changes in log concentrations at any stage were estimated, by employing Markov chain Monte Carlo (MCMC) sampling. Thus *in vitro* infectivity within and between strains can be quantified, separating biological variability from experimental uncertainty.

The sum of all log changes in concentration of *Salmonella* (ml^−1^) throughout the GIT system from ON up until concentration invasion into Caco-2 cells (INV) was defined as log P(inf), an estimate for *in vitro* infectivity. The mean P(inf), including a 95% credible interval (C.I.), was calculated for each strain tested in the GIT system. In addition, a mean P(inf), standard deviation (SD) and 95% C.I. were calculated at the serovar level averaging posterior samples of multiple individual strains when available (Table 1).

### Whole Genome Sequencing (WGS)

#### DNA Isolation

Genomic DNA from studied strains was extracted using the MagAttract HMW DNA kit (Qiagen, Venlo, Netherlands) according to the manual “Purification of High Molecular Weight Genomic DNA from Gram Positive Bacteria” (including a lysozyme step). An extra concentration and wash step were included to increase the yield and purity of DNA (20–50 ng/μl).

The concentration and purity of the DNA were measured using a Quantus device (Promega, Leiden, Netherlands) according to the manual for using the QuantiFluor dsDNA kit (Promega).

#### Next Generation Sequencing (NGS)

Paired-end sequence reads were generated using the Illumina HiSeq 2500 system with standard Illumina filtering and quality control. FASTQ sequence files were produced using the Illumina Casava pipeline version 1.8.3.

#### Processing Sequence Reads

For downstream bioinformatic analyses, sequence reads were assembled using SPAdes (Bankevich et al., 2012) on the ARIES platform of the public Galaxy server at Istituto Superiore di Sanita^2^. The settings of the SPAdes assembler were; Run only assembly (without read error correction): No, Careful correction: Yes Automatically choose k-mer values: No, K-mers to use, separated by commas: 21, 33, 55, Coverage Cutoff: Off.

Quality of resulting assemblies was assessed by checking the total number the contigs, their maximum and average length, and N50 per strain (Supplementary Appendix A).

The sequence data of the isolates are available. Accession numbers are provided in Supplementary Appendix A.

#### Phylogenetic Analysis

The read-based, reference-free method kSNP (Gardner et al., 2015), a k-mer based approach for detecting single-nucleotide variants, was used for phylogenetic analyses. kSNP3 was applied via the Galaxy project Aries (see footnote 1). The kmer starting value was set at a lower limit of 19 and a final upper limit of 191 with a search step of 2.

#### Virulence Genes

Sequences from virulence genes listed in the *Salmonella* section of the virulence factor database^3^ and some additional ones found in the literature were downloaded (e.g., Bolton et al., 2012; Suez et al., 2013; Khoo et al., 2015). These conformed a set of 233 putative virulence genes.

The tested virulence gene sequences (chromosome and plasmid) were selected for *S.* Typhimurium LT2, *S*. Enteritidis P125109, *S*. Agona SL483, *S*. Heidelberg SL476 and *S*. Typhi CT18. The investigated virulence genes are given in Supplementary Appendix B.

The presence or absence of the 233 virulence genes/factors was investigated by individual BLASTn analysis in the “Align two or more sequences” option^4^ of the assembled NGS data. The algorithm parameters were set to: expect threshold 10; word size 28; max matches in a query range 0; match/mismatch scores 1, −2; gap costs linear. Genes were marked as present if the BLAST showed an alignment of sequences of a strain with >90% identity and a coverage of >95% (Laing et al., 2017).

#### Epidemiological Surveillance Data

Notification data for the *Salmonella* serovars used in this study were obtained from the national laboratory surveillance of RIVM (Zomer et al., 2014).

#### Linking *in vitro* Virulence to the Presence/Absence of Virulence Genes

A description of the thorough Bayesian statistical analysis of the *in vitro* virulence of the 59 *Salmonella* strains has been described in section “GIT System Data Analysis” with further reference to Wijnands et al. (2017). Based on these analyses, accounting for uncertainty in P(inf) estimates, the *Salmonella* strains could be grouped into four categories: Low: P(inf) < 10^−3^, mid-Low: 10^−3^ < P(inf) < 10^−2^, mid-High: 10^−2^ < P(inf) < 10^−1^ and High: P(inf) > 10^−1^. Subsequent descriptive statistics, simple linear regression and principal component analysis (details provided in Supplementary Appendix C) were used to visualize potential relations between the 233 investigated virulence genes and a variety of phenotypic characteristics: survival through the separate stages of the GIT system, inferred from the Bayesian statistical analysis of *in vitro* virulence, and human cases of illness.

## Results

### *In vitro* Virulence

**FIGURE 2:**
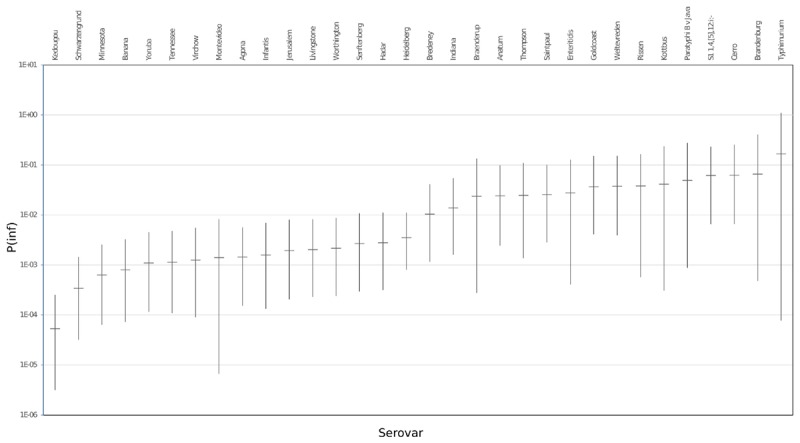
P(inf) at serovar level from low (left) to high (right) infectious serovars in the GIT system. The horizontal lines indicate the mean P(inf) and the bars represent the 95% C.I. See Table 1 for the associated values.

**FIGURE 3:**
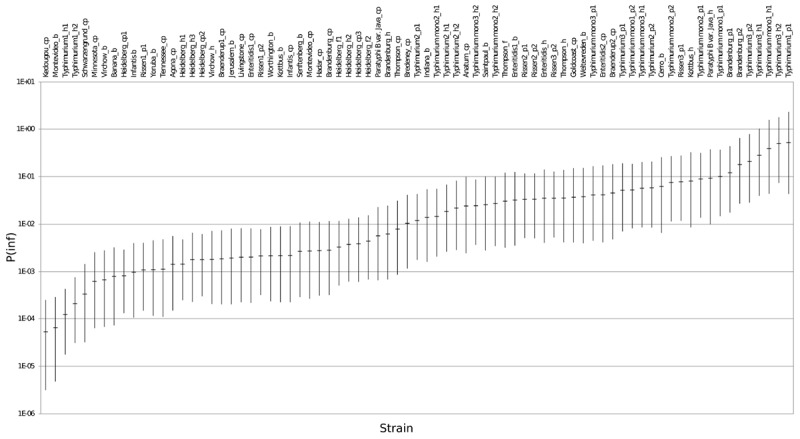
P(inf) for all individual test strains from low (left) to high (right) infectious strains in the GIT system. The horizontal lines indicate the mean P(inf) and the bars represent the 95% C.I. See Table 1 for additional strain information (b, broiler; cp, chicken product; f, food; h, human; p, pork). Strains of the same serovar and source have equal indicators (e.g., Typhimurium1_cp). Replicates of the same strain are numbered as indicated by the underscore (e.g., _cp1).

The mean *in vitro* virulence P(inf) with 95% credible intervals (C.I.) calculated from the GIT experiments were sorted by average P(inf). Figure 2 shows P(inf) posteriors calculated for strains grouped at serovar level and Figure 3 shows P(inf) posteriors calculated for all individual strains. The associated values together with the standard deviation of the P(inf) posterior distributions on serovar level are provided in Table 1. Variability exists both within and between serovars. The average P(inf) ranges in the order of 1E-05 to 1E-01 for individually tested strains in the GIT system. A *Salmonella* Kedougou strain had the lowest P(inf), 5.3E-05 for an individual strain while a *S*. Typhimurium strain had the highest individual P(inf), 5.2E-01 and on average 1.7E-01 on serovar level. Individual strains of *S.* Typhimurium cover, however, a broad range of *in vitro* virulence; 1.2E-04 for one of the human strains and from 1.2E-02 up to 5.2E-01 for the other strains.

Figure 3 shows that the 95% C.I. describing the experimental P(inf) uncertainty were within the same range for individual strains. The 95% C.I.’s were larger at serovar level for some serovars compared to the 95% C.I.’s of the underlying strains. This indicates a larger variability in P(inf) for individual strains of the same serovar compared to those of other serovars was observed. For example, *S.* Montevideo showed a relatively large 95% C.I. which can be explained by the relatively large difference in P(inf) for the individual strains, one broiler strain with a P(inf) of 6.5E-05 and one chicken product strain with a P(inf) of 2.7E-03. On the other hand, *S.* Infantis showed a relatively small 95% C.I. at serovar level with both broiler and chicken product strains. *S.* Heidelberg also showed a relatively small 95% C.I. at serovar level. This is caused by a relatively large number of replicates with three test strains and a narrow range in mean P(inf): 8.3E-04 – 4.4E-03 for the individual test strains. The food (f) and human (h) *S.* Heidelberg strains stem from the same outbreak, and the chicken product (cp) strain was not related to the outbreak, but still had a comparable P(inf).

All replicate experiments using the same *Salmonella* strain had overlapping 95% C.I.’s. For example, *S.* Rissen consists of three pork strains that have all been tested twice, expressed as Rissen(1) with a P(inf) of 1.1E-03 and 2.1E-03, Rissen(2) with both a P(inf) of 3.3E-02 and Rissen(3) with a P(inf) of 3.5E-02 and 7.8E-02 (Figure 3).

Overall, Figure 2 shows variability in P(inf) between different *Salmonella* serovars, where *S.* Kedougou is characterized by the lowest P(inf) (<1E-04), followed by a group with a P(inf) in the range of 1E-03 -1E-02 and a group with a P(inf) > 1E-02. The second group contains one serovar (*S.* Infantis) often associated with human cases of illness, whereas the third group contained Typhimurium and Enteritidis which have the highest human disease incidence. However, also serovars with low incidence (for example Gold Coast and Kottbus) can be found in highest range of P(inf) and large variability in P(inf) can be seen between individual *Salmonella* strains (Figure 3).

### Virulence Genes

Among the 233 investigated virulence factors, variability in presence/absence in the strains was concentrated to 101 genes, because 14 were absent and 118 were present in all tested strains (see Supplementary Appendix B). A gene was assigned “present” when it was detected in >70% of the tested strains for each serovar. The five serovars with the highest number of virulence genes in individual strains were: Heidelberg (181), Typhimurium and Enteritidis (both 179), Virchow (177) and Thompson (176). The five serovars with the lowest number of virulence genes in individual strains are: Schwarzengrund (151), Bredeney (150), Cerro (148), Montevideo (144), and Brandenburg (142).

**FIGURE 4:**
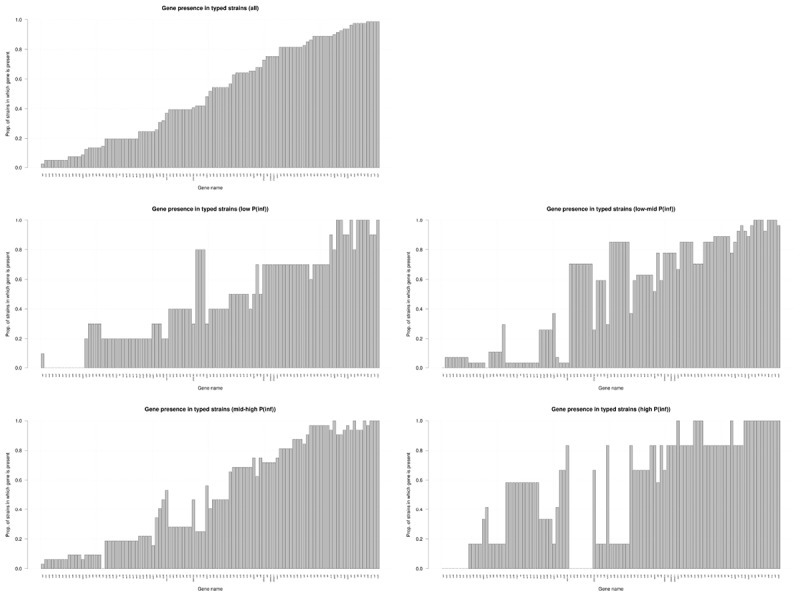
Presence/absence of virulence genes in all strains investigated (*n* = 59); low: P(inf) < 1E-03 (*n* = 8), low-mid: 1E-03 < P(inf) < 1E-02(*n* = 22), mid-high: 1E-02 < P(inf) < 1E-01(*n* = 23), high: P(inf) > 1E-01 (*n* = 6). Genes that were either present or absent in all strains are not shown.

Figure 4 shows the presence of the 101 determinant virulence genes for all strains and for those related to the different categories (low, low-mid, mid-high, and high) of the *in vitro* virulence of the GIT system. There is no straight forward association between gene presence and P(inf) when comparing gene presence in all the categories with the gene presence for each category separated, especially those in the extremes, whose presence is either close to a value of 1 or 0. Only a few genes are evidently increasing in presence as the P(inf) increases to higher categories (*safA*, *gogB*, *ssel.srfH*, STM1043, *sodCI*, *stdA*, *sseK2*, and the *sef* operon) and another few are increasing in absence as the P(inf) category increases (the *stk* operon and the *ste* operon).

**FIGURE 5:**
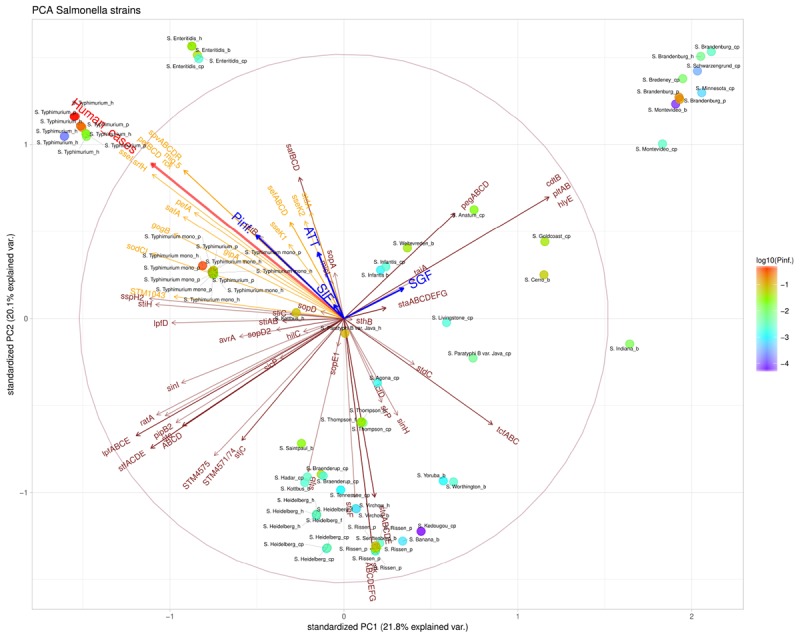
Principal component analysis (PCA) scatterplot calculated from the presence/absence of the various virulence genes (brown and orange arrows) in the *Salmonella* strains under investigation. The number of human cases (shown in red arrow) and survival up to the indicated stages SGF, SIF. ATT and INV [indicated as P(inf)] (shown in blue arrows). The color scale of the dots characterizes P(inf) of individual strains (indicated in the legend). Orange virulence genes are those associated with a positive P(inf) effect (*p*-value < 0.05) (see section “Genetic Variability Linked to *in vitro* Virulence,” Table 2). The length and direction of the variable arrows indicate how those variables contribute to the separation of the data when projected on the two components which explain the most data variance, *i.e.*, PC1 and PC2. An arrow reaching the large circle means that it is fully projected on the two principal component plane. See Table 1 for additional strain information (b, broiler; cp, chicken product; f, food; h, human; p, pork). Strains of the same serovar and source have equal indicators (e.g., Typhimurium1_cp).

**FIGURE 6:**
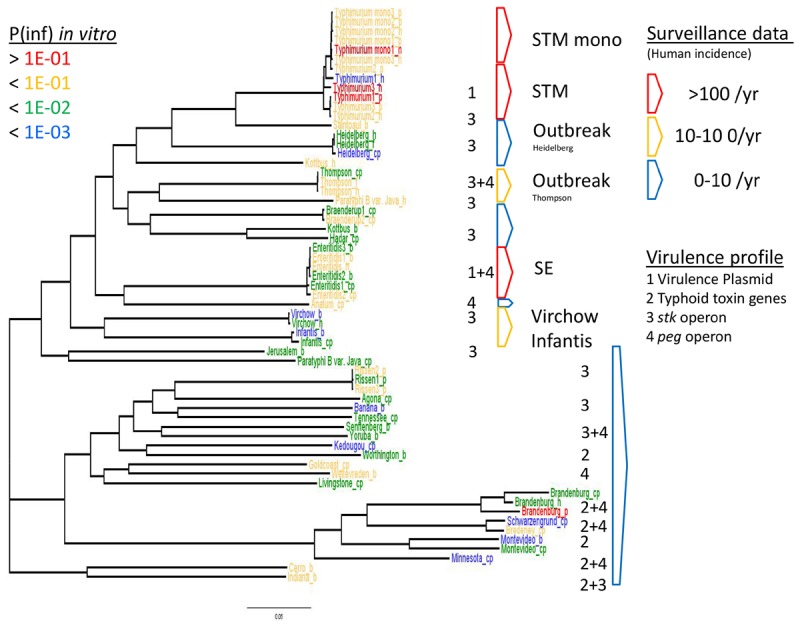
WGS based phylogeny related to *in vitro* virulence of the GIT system (in this study) and surveillance data. The serovar names of the *Salmonella* strains with the highest P(inf) are written in red (>1E-01), mid-high (<1E-01) in yellow, low-mid (<1E-02) in green and the lowest in blue (<1E-03). The *Salmonella* top five serovars related to human cases are marked in red (>100/year) and yellow (10–100/year), the lowest number of human cases (0–10/year) are blue (Zomer et al., 2014). See Table 1 for additional strain information (b, broiler; cp, chicken product; f, food; h, human; p, pork). Strains of the same serovar and source have equal indicators (e.g., Typhimurium1_cp). Numbers 1, 2, 3, and 4 indicate the virulence profiles found as clusters in the PCA analysis, present in each strain and grouped by serovar.

The presence/absence of virulence genes on strain level was submitted to a principal component analysis (PCA, Figure 5). Only those 101 virulence genes that showed variability in their presence/absence among the strains were included. Based on the PCA analysis, different clusters could be identified based on the presence of virulence genes, which could be grouped under different virulence profiles (Figure 6). Profile 1, the virulence genes located on a plasmid; *mig*-5 and operons of *pef, rck* and *spv* were only present in serovars with a high P(inf), *S.* Typhimurium and *S.* Enteritidis. Profile 2, the typhoid toxin genes *cdtB*, *hlyE, plt*, and *taiA* operon were only found in the *Salmonella* serovars Brandenburg, Bredeney, Gold Coast, Indiana, Minnesota, Montevideo, and Schwarzengrund. Profile 3, the *stk* operon virulence genes were present in the outbreak strains of *S*. Heidelberg and *S.* Thompson, but also in serovars like Banana, Braenderup, Hadar, Indiana, Paratyphi B var. Java (cp), Rissen, Saintpaul, Senftenberg, Virchow, Worthington and Yoruba. And profile 4, the *peg* operon fimbrial virulence genes were not only present in one of the most relevant serovars from an epidemiological viewpoint, *S*. Enteritidis, but also in serovars; Anatum, Brandenburg, Bredeney, Livingstone, Minnesota, Schwarzengrund, Thompson, Worthington and Yoruba.

### The GIT System

Figure 5 shows that stomach survival (shown as SGF) was positively associated with the presence of Typhoid toxin genes. Survival through other stages of the GIT system [shown as SIF, ATT, and P(inf), respectively] were associated with higher human disease incidence and more specifically, with *S*. Typhimurium, *S.* Enteritidis and monophasic *S*. Typhimurium.

### Genetic Variability Linked to *in vitro* Virulence P(inf)

The distribution of the presence/absence of genes among strains with a relatively high P(inf), > 1E-01 was compared to strains with a relatively low P(inf), <1E-03 (Figure 4). Highly *in vitro* infectious strains did not possess any of the *stk* genes. Genes associated with highly *in vitro* infectious strains were *gogB, mig*-5*, safA*, STM1043 and operons *pef, rck* and *spv.*

A simple linear regression model (Pielaat et al., 2015) was applied in an attempt to identify those genes which could be associated with high P(inf) (Table 2). All plasmid related genes (*mig*-5 and operons of *pef*, *rck* and *spv*) were part of this group and were only present in the human cases associated serovars Typhimurium and Enteritidis. All the virulence genes present in more than 50% of the strains with the highest P(inf) (*gogB, mig*-5, *pef*, *rck*, *safA*, *spv*, and STM1043) are mentioned in Table 2 as the genes that have a positive association with P(inf). The results from the linear regression model were used as an identification tool rather than for testing an hypothesis on whether the studied genes are responsible for high P(inf) values. Results suggest that not all strains with a relatively high P(inf), > 1E-02, possessed the identified virulence genes having a gene effect with *p*-value < 0.05 (marked with an asterisk in Supplementary Appendix B). For example, *S.* Rissen with a comparable P(inf) to *S.* Enteritidis [P(inf) of 3.8E-02] possesses none of the 19 virulence genes with *p*-value < 0.05, while 13 of these genes were present in *S.* Enteritidis (only *gipA*, *gogB*, *pefA*, *safA*, *stdA*, and STM1043 were absent).

**Table 2:** Parameter values of the gene effect (*n* = 19) with a *p*-value < 0.05 which are positively associated with P(inf).

Name	Gene effect	*p*-value	Biological relevance	Site
*gipA*	0.054	0.03429	Specific virulence factor	Phage
*gogB*	0.088	0.00016	Translocator effector protein	Phage
*mig*.5	0.100	0.00059	Carbonic anhydrase, macrophage inducible gene	Plasmid
*pefA*	0.146	0.00001	Fimbrial operon PEF	Plasmid
*pefBCD*	0.100	0.00059	Fimbrial operon PEF	Plasmid
*rck*	0.100	0.00059	Attachment/invasion locus protein precursor, serum resistance	Plasmid
*safA*	0.089	0.00017	Outer membrane protein	Fimbriae
*sodCI*	0.055	0.01386	Superoxide dismutase, stress protein	Phage
*spvRABCD*	0.100	0.00059	Growth in host cells	Plasmid
*sseI.srfH*	0.083	0.00029	Translocator effector protein	Chromosome
*sseK2*	0.130	0.00359	Translocator effector protein	Chromosome
*stdA*	0.059	0.00834	Fimbrial major subunit	Chromosome
STM1043	0.060	0.00828	Attachment/invasion protein	Chromosome

*The genes with the same p-value and from the same operon are merged.*

### Phylogeny Related to *in vitro* Virulence and Surveillance Data

The relation of the phylogenetic tree based on the WGS data of all tested *Salmonella* strains to *in vitro* virulence expressed in different P(inf) categories, showed no clear phylogenetic relationship regarding virulence (Figure 6). Serovars with a P(inf) > 1E-02 show a tendence to be clustered [(monophasic) Typhimurium, the outbreak strains (Thompson and Heidelberg) and Enteritidis]. The relation of human salmonellosis surveillance data on serovar level of the Dutch population (Zomer et al., 2014) to the phylogenetic data suggests that serovars with low number of human cases (0–10 per year) in the same part of the phylogenetic tree (e.g., Rissen and Brandenburg serovars in Figure 6). There was no apparent relation between the serovars possessing the same virulence profiles to the phylogenetic tree. An exception were the serovars with typhoid toxin genes virulence profile, which most could be located in the same part of the phylogenic tree (Figure 6).

## Conclusion

The virulence of in total 59 strains of 32 different *Salmonella* serovars was assessed in an *in vitro* gastrointestinal tract system including attachment and invasion into cultured human intestinal epithelial cells. Statistical association with the presence/absence of 233 virulence genes in these strains was studied, and the results compared with surveillance data of human salmonellosis.

The *in vitro* infectivity estimate (P(inf)) was quantified and strains were differentiated based on their variability in P(inf).

Although many of the human associated serovars (e.g., *S.* Typhimurium and *S.* Enteritidis) clustered in the relatively high P(inf) group, still, large variability in P(inf) could be seen between individual *Salmonella* strains.

Based on sequence data, it was possible to cluster serovars with the same profile of virulence genes (virulence plasmid, Typhoid toxin, *peg* operon and *stk* operon.) but there was no clear association with phenotypic virulence P(inf). Overall, individual strains showed larger variability in P(inf) in association with virulence genes than could be explained by serovar alone. Ranking a public health risk of different *Salmonella* using point estimates in *in vitro* P(inf) on serovar level alone without considering biological variability of the underlying strains is, therefore, not recommended.

Finally, we would like to stress that a systematic approach in which molecular data is linked to other strain characteristics is a crucial step for future MRA studies. The aggregation of molecular data based on biologically relevant information will decrease the uncertainty in microbiological risk assessments. It will also prevent the initiation of a disaggregation problem in which the number of hazards will increase exponentially when a human risk assessment is based on the analysis of molecular data alone and each strain forms a distinct hazard.

## Discussion

Traditional MRA methodologies based on the Codex principles for risk analysis (Codex Alimentarius Commission [CAC] (1999)) are currently revised by the scientific community in the light of the increasing amount of available molecular data. Pielaat et al. (2015) suggested that the integration of one dimensional *in vitro* testing (*e.g.*, invasion into Caco-2 cells) with multiple dimensional interpretation of effects on a molecular level is a step forward to solve the mapping problem of translating this high dimensional information on molecular level to a single measure of risk (*e.g.*, number of ill cases). In this study, we used descriptive statistics, simple linear regression and principal component analysis to visualize this integration of *in vitro* testing (passage through the GIT system) with human cases of illness and 233 investigated virulence genes. This type of analysis enabled us to identify a clustering of potentially relevant biological associations between phenotypic and genotypic data for microbiological risk assessment of *Salmonella*. This approach forms the basis to ultimately be able to build a valid, non-overparameterized, statistical model to identify marker genes for risk assessment.

In this study biological variability in *in vitro* infectivity was integrated with genetic diversity and human salmonellosis surveillance data to reveal potential new insights in the public health risk of different *Salmonella* strains. Results show (Figure 3) that *in vitro* infectivity, P(inf), for human *Salmonella* strains within one serovar is higher than for strains from other non-human sources within the same serovar [except for *S.* Typhimurium with high variability among all individual strains and monophasic *S.* Typhimurium, variable but all strains have a relatively high P(inf)]. This is in accordance with an assumption by Oliveira et al. (2011) that the history of a strain may have influence on its virulence. Further research would be needed with more strains per serovar to quantify specific within and between *in vitro* variability for all individual serovars as used in this study. However, concluding that the *in vitro* P(inf) variation among individual strains from the same serovar is larger than the P(inf) variation found between serovars still holds. This because the unequal number of strains per serovar has been already accounted for by the presented 95% credible intervals, which represent uncertainty on the true P(inf) at serovar level. Moreover, our experiments show a gradual range in *in vitro* virulence from 10^−5^ to 10^−1^. It represents the minimal range for measuring *in vitro* virulence as input for biologically relevant risk assessment for *Salmonella.* Including more strains will not necessarily change the overall conclusions.

The P(inf) agrees with human surveillance data where serovars with a high P(inf) have a high number of reported human cases [*S.* Typhimurium and the monophasic *S.* Typhimurium both with the highest average P(inf) and number of human cases, followed by *S.* Enteritidis (see Figure 6)]. *S.* Infantis and *S.* Virchow are an exception with a relatively low P(inf), but still between 10–100 confirmed human cases (Zomer et al., 2014). Strains associated with outbreaks caused by *S.* Heidelberg and *S.* Thompson from both food and human origin have comparable P(inf). In both cases, the P(inf)s of the outbreak-related strains are higher than the P(inf) of non-outbreak related strains originating from chicken products. The outbreak-related strains *S*. Heidelberg and *S*. Thompson (Friesema et al., 2014; van Rijckevorsel et al., 2015) did not show any specific association with survival through the stages in the GIT system (Wijnands et al., 2017; Figure 5). Of course, in this study we only consider *in vitro* P(inf) as explanatory variable for human cases, whereas other pathogen/host factors like prevalence, growth potential, acquired immunity, etc., influence the number of human cases.

Among the 233 investigated virulence genes, 101 showed variability in presence/absence in the *Salmonella* strains (see Supplementary Appendix B). *In vitro* P(inf) is slightly positively associated with the reported number of *S*. Typhimurium and *S*. Enteritidis human cases and specific plasmid related virulence genes (*mig*-5, operons of *pef*, *rck* and *spv*). However, not all serovars with a relatively high P(inf), > 1E-02, are associated with the identified virulence genes having positive association with P(inf) (gene effect with *p*-value < 0.05). More strains with an equal distribution over different serovars would need to be investigated to make any further statements about phenotypes and associated genes inside serovars. Moreover, a pangenomic approach on a larger strain set representing the total (molecular) variability of *Salmonella* would increase the probability to detect genes highly associated with P(inf).

The presence of virulence genes among different *Salmonella* strains as investigated in this study (see Figure 5 and Supplementary Appendix B) can be discussed against other published studies with a main focus on those genes identified to be potentially positively associated with P(inf) (see Table 2). These genes or operons will be addressed below one by one:

- The *gipA* gene is involved in Peyer’s patch survival and it concurs to *Salmonella* colonization of the M cells in the Peyer’s patch (Stanley et al., 2000). In our study *gipA* was present in strains of *S*. Typhimurium (h and p), monophasic *S*. Typhimurium (h and p), *S*. Virchow (h and p), *S*. Brandenburg (cp), and *S*. Weltevreden (p). Huehn et al. (2010) showed comparable results with the *gipA* gene being present in a part of the tested Typhimurium and Enteritidis strains and almost all Virchow strains. Beutlich et al. (2011) found the *gipA* gene in one Typhimurium strain.

- The gene *sodC1* (superoxide dismutase), a virulence associated stress protein located on prophage, was present in *S*. Typhimurium (h and p), *S*. Heidelberg (cp, f and h), *S*. Enteritidis (cp, h and p), monophasic *S*. Typhimurium (h and p), *S*. Weltevreden (p) and absent in all strains with a low P(inf). Huehn et al. (2010) and Osman et al. (2014) also showed presence of *sodC1* in *S*. Typhimurium and *S*. Enteritidis.

- The *spv* (*Salmonella* plasmid virulence) operon was found to be associated with *Salmonella* survival and growth in macrophages by Rychlik et al. (2006), and appeared to be only present in serovars Typhimurium and Enteritidis in our study. Huehn et al. (2010), Guedda et al. (2014), and Hoffmann et al. (2014) all showed comparable results with the *spv* operon being present in Typhimurium and Enteritidis, but absent in other serovars under investigation.

In addition to the relation of our findings compared to those in other studies concerning the genes in Table 2, the following remarkable results were observed:

- The *staA* gene, originally identified uniquely in serovar Typhi (Townsend et al., 2001; Al-Dahhan et al., 2015), was now found to be present in four of the investigated strains all isolated from a chicken product: *S.* Agona, *S.* Anatum, *S*. Livingstone, and *S.* Gold Coast. Huehn et al. (2010), did not find the *staA* gene in any of the 77 investigated strains [serovars Enteritidis (19), Hadar (14), Infantis (11), Typhimurium (21), and Virchow(11)].

- The *stk* fimbrial operon was present in multiple serovars, but remarkable was that the *stk* genes were present in all the investigated Heidelberg and Thompson including outbreak strains. However, Foley et al. (2011) also mentioned the presence of *stk* genes in isolates of *S*. Heidelberg but indicated that genes were differentially expressed among the *S*. Heidelberg strains examined.

- Typhoid toxin genes known from *S*. Typhi CT18 (*cdtB, hlyE*, and *pltAB*) were present in 10 strains, among the serovars Brandenburg, Bredeney, Gold Coast, Indiana, Minnesota, Montevideo and Schwarze Grund while *taiA* (Typhi-associated invasin A) was only present in Gold Coast, and Minnesota. The presence of *cdtB* and *pltAB* in isolates of Montevideo, Schwarzengrund, Bredeney was also found by Suez et al. (2013). Fuentes et al. (2008) found that *hlyE* (pore-forming hemolysin) mutants show impaired invasion of human epithelial cells *in vitro*, and its expression in *S*. Typhimurium improves the colonization of deep organs in mice, demonstrating that the HlyE hemolysin is a virulence determinant.

- The *peg* operon showed to be present in most important serovars from an epidemiological viewpoint. The role of *peg* operon in colonization and virulence has been established in a number of model experiments (Silva et al., 2012).

Most of the virulence genes that have a positive association with P(inf) were plasmid and phage associated genes (Table 2). Cheng et al. (2015) mentioned that the disease incidence of poultry strains in humans may be attributable to the distribution of phage-associated virulence genes *sodCI, sopE*, and *sseI* and the plasmid located *spvB* in *S*. *enterica* serovars.

Some differences were observed in the possession of virulence genes in strains of Typhimurium and monophasic Typhimurium, both with a high P(inf) and high incidence in the surveillance data. Most remarkable was the absence of plasmid related virulence genes (*mig*-5 and *pef, rck* and *spv* operons) in all the monophasic Typhimurium strains and also in one Typhimurium strain (still with a high P(inf)). There was more variation in the number of virulence genes within the Typhimurium strains (169–179 of the 233) compared to monophasic Typhimurium strains which all possess the same total (169 of the 233). García et al. (2014) reported that most isolates of Typhimurium strains contained a virulence plasmid but they did not find it in isolates belonging to the monophasic variant. Still, it should be noticed that currently applied fragmented assembly procedures may induce false negative results for the absence of genes screened by blast. However, the probability of having false negative results will be limited in our study. An additional screening on virulence genes partly present on two different assemblies has been performed and still included as a positive result in further analysis.

Independent of serovar, an association was shown between certain virulence genes in the strains and their in *vitro* virulence (Figure 4). Further in depth investigation is needed to assess how much of the variability in P(inf) in the in *vitro* GIT system can be explained by one or a combination of virulence genes as identified in this study. This would involve the phenotypic analysis of a larger set of strains representing the whole (molecular) *Salmonella* population resulting in sufficient power to build a valid statistical model for causal analysis. Finally, biological confirmation (e.g., with gene knock-out experiments) and reproducibility of the analysis is a prerequisite to confirm any causal relationship between genotypic and phenotypic characteristics.

## Author Contributions

AK was in charge of all practical work concerning the NGS data and GIT system lab analysis and NGS analysis, data interpretation, and the design, and data generation during the whole study. ABM developed together with AP the latest Bayesian statistical framework for the calculations on the GIT system, and is responsible for statistical calculations under that framework and for part of the results from the NGS data and PCA analysis. LW was responsible/supervisor for all the work on the GIT system. ED-v was responsible for the practical lab work on the strains and the GIT system. AvH was supervisor for all the work on the obtained NGS data. AP and EF planned and designed the whole project. AP was project-leader and responsible for the study design, the statistical calculations and supervision of finance. AK and AP have written the manuscript. All authors read, commented, and approved the final manuscript.

## Conflict of Interest Statement

The authors declare that the research was conducted in the absence of any commercial or financial relationships that could be construed as a potential conflict of interest.

## Footnotes

1 http://ilsi.eu/task-forces/food-safety/microbiological-food-safety/

2 https://w3.iss.it/site/aries

3 http://www.mgc.ac.cn/VFs/main.htm

4 https://blast.ncbi.nlm.nih.gov/Blast.cgi
