# Supplementary material for: Phenotypic Prediction: Linking in vitro Virulence to the Genomics of 59 Salmonella enterica Strains
Source: Front Microbiol. 2019 Jan 9;9:3182. doi: 10.3389/fmicb.2018.03182 (PMC6333659; doi:10.3389/fmicb.2018.03182)
Supplement: Supplementary file 3 [file Data_Sheet_1.pdf]

Appendix C: Details on linear regression and principal component analyses.

The linear regression and principal components analyses were carried out using the statistical programming language R, version 3.4.3, and its package "stats", which is standard to the distribution. Both analyses considered as input data the average P(inf) for each strain, computed under the Bayesian statistical framework described by Wijnands et al. (2017), where biological and experiment variability are taken into account by inferring them from the data from all strains.

The linear regression analysis was carried out by using the function `lm` for fitting linear models from, where the contribution of each gene's presence was independently tested against the average P(inf), for all the strains studied. We considered only those genes that were not present or absent in all studied strains.
Therefore, per gene of interest, the linear model results were calculated using the statement

```
Lm.result <- lm(P.inf ~ gene)
```

Where `P.inf` is a vector containing the average P(inf) for every strain, and `gene` is a vector containing the presence of the gene of interest for every strain with values 0 and 1.

The principal component analysis was carried out by inputting in the function `prcomp` an array of data which for every studied strain included its average P(inf), presence of each gene, and number of human cases if the strain was related to an outbreak.
